# Burden, Depression, and Awareness of Information on Safety Behavior in Korean Hemodialysis Patients during the COVID-19 Pandemic

**DOI:** 10.3390/ijerph181910348

**Published:** 2021-09-30

**Authors:** Ok-Hee Cho, Yun-Hee Cho, Mi-Young Chung

**Affiliations:** 1 Department of Nursing, College of Nursing and Health, Kongju National University, 56 Gongjudeahak-ro, Gongju-si 32588, Chungcheongnam-do, Korea; ohcho@kongju.ac.kr; 2 Department of Nursing, Daejeon Sun Hospital, 29 Mokjung-ro, Jung-gu, Daejeon 34811, Korea; choryu1128@hanmail.net; 3 Department of Nursing Science, Sunmoon University, 70 Sunmoon-ro 221beon-gil, Tangjeong-myeon, Asan-si 31460, Chungcheongnam-do, Korea

**Keywords:** pandemic, COVID-19, renal dialysis, health behavior, information-seeking behavior

## Abstract

The purpose of this study was to investigate the relationships among burden, depression, awareness of information (AIC), and safety behavior among hemodialysis patients in Korea during the COVID-19 pandemic. The study participants included 149 patients who received hemodialysis at seven general hospitals in Korea between January and February 2021. A structured questionnaire was used to survey their levels of burden, depression, AIC, adherent safety behavior (ASB), and dysfunctional safety behavior (DSB). The study results showed that the influencing factors of ASB for COVID-19 were AIC (*β* = 0.265, *p <* 0.001), the burden of “not receiving hemodialysis on time” (*β* = 0.233, *p* = 0.008), and the burden of “social exclusion of hemodialysis patients” (*β* = 0.186, *p* = 0.032). The influencing factors of DSB were the burden of “social exclusion of hemodialysis patients” (*β* = 0.258, *p =* 0.003) and AIC (*β* = 0.217, *p =* 0.004). As the COVID-19 pandemic continues, the latest evidence-based information must be provided to hemodialysis patients to promote self-care and prevention behavior that encourages ASB and discourages DSB.

## 1. Introduction

Since the first report of a serious respiratory syndrome associated with severe acute respiratory syndrome coronavirus, which became known as coronavirus disease (COVID-19), in Wuhan, China, in October 2019, lives have been threatened across the globe. As of July 2021, 184,324,026 confirmed cases of COVID-19 and 3,992,680 deaths had been reported worldwide [1]. Since the first reported case in January 2020 to June 2021, Korea has reached 153,659 confirmed cases and 2034 deaths [2]. Symptoms associated with COVID-19 are primarily respiratory symptoms, but the disease may also affect various other organs, including the kidneys and the heart, as well as the digestive, circulatory, and nervous systems [3]. Patients with underlying diseases such as hypertension, diabetes, cardiovascular disease, respiratory disease, and cancer are more vulnerable to COVID-19 and, when diagnosed with COVID-19, have a poorer prognosis than healthy individuals [4].

Patients with end-stage renal disease have irreversible damage to their kidney function and depend on kidney replacement therapy to sustain their lives. In Korea, 75.1% of end-stage renal disease patients receive hemodialysis therapy [5]. Most patients with end-stage renal disease have underlying conditions such as hypertension and diabetes, and have suppressed immune systems, which makes them more vulnerable to infection [6]. Previous studies have reported that patients on hemodialysis who were infected with COVID-19 had higher rates of complications such as shock, acute respiratory syndrome, arrhythmia, and acute cardiac injury, with a distinctly higher mortality rate among infected hemodialysis patients (13%) compared to that of the general population (4%) [7,8].

Even during a pandemic, hemodialysis patients must visit a medical institution two to three times a week and have close contact with others in order to undergo hemodialysis [5]. Moreover, these patients experience uremia symptoms such as fever and fatigue, which often appear before and after hemodialysis but that can also be confused with COVID-19 symptoms, making early COVID-19 diagnosis difficult [7]. These vulnerabilities may increase fear and anxiety in hemodialysis patients, leading to adherent safety behavior (ASB). This includes safety behaviors such as practicing hand hygiene, wearing facial masks, and social distancing (e.g., reducing the use of public transport, avoiding crowded places, and postponing or canceling social events) [9,10].

Various governments have mandated mask wearing and implemented contact bans and closures to contain the spread of COVID-19. It is socially desirable for individuals within a society to implement ASB. However, dysfunctional safety behavior (DSBs) such as panic buying and hoarding prioritize self-interest, in that they restrict other people’s purchases by monopolizing limited resources. DSBs are a coping action to relieve fear and anxiety about the current situation and to regain a sense of control [11,12]. It can therefore be observed that socio-psychological factors influence ASB and DSB.

Studies have reported that the general public demonstrates greater compliance with infection prevention rules such as handwashing, mask wearing, and social distancing, and seek more information and knowledge when they have greater fear and a more acute awareness of COVID-19 [13,14]. A study of the general public reported that an accurate awareness of COVID-19 increased ASB, while low levels of knowledge and situational awareness were responsible for DSBs such as hoarding [15,16]. Additionally, trust in society and a sense of responsibility as a member of society were found to be related to ASBs [17].

During the H1N1 influenza pandemic in 2009, public concern about infection led to the adoption of ASB due to an increasing number of public health officials promoting information-seeking behavior [18]. Those with accurate information showed a higher level of knowledge about the H1N1 flu, and were actively using personal hygiene products such as masks and hand sanitizers, and showed a high willingness to vaccinate [19]. In the current pandemic, individuals must accurately recognize the changing public health messages [12]. Many vulnerable populations are further alienated by inappropriate health communication, which can subsequently pose significant risks to themselves and the community. Patients with comorbidities had low knowledge of COVID-19 and decreased ASB compliance, despite the risk of infection [12,18]. It is necessary to understand the impact of information awareness and psychological vulnerability on the implementation of safety behaviors in hemodialysis patients in order to help them safely withstand the pandemic.

Most of the current studies related to COVID-19 have focused on epidemiology [20], the clinical course of the disease [21], the development and efficacy of vaccines [22], and infection control guidelines [23]. Some studies have reported psychological problems such as depression, anxiety, and fear, and have focused on the general public and on patients with autoimmune disease, cancer, and chronic disease [9,24,25,26]. However, studies focused on safety behavior geared toward preventing risk are lacking. In particular, research on hemodialysis patients who are vulnerable to infection due to disease, treatment, and environmental factors is insufficient. Accordingly, this study aimed to identify factors that can promote safety behaviors that can prevent infectious disease among hemodialysis patients within the current pandemic situation and establish basic data for the development of infectious disease control interventions.

## 2. Methods

### 2.1. Study Design

This study was designed as a cross-sectional survey, utilizing a questionnaire focused on burden, depression, AIC, and safety behavior among hemodialysis patients.

### 2.2. Participants and Data Collection

The study participants were patients receiving hemodialysis at seven general hospitals in Korea. The inclusion criteria were:

1. Adults aged 20 years or older;
2. Individuals with no history of COVID-19 infection;
3. Patients receiving hemodialysis therapy 2–3 times per week;
4. Patients receiving hemodialysis therapy during outpatient visits to the renal dialysis unit;
5. Individuals with no difficulties understanding Korean or communicating.

The exclusion criterion was receiving hemodialysis therapy as an inpatient. The sample size needed for the regression analysis was calculated using the G*Power 3.1.9.4 program. Based on 13 predictor variables, a significance level of 0.05, a medium effect size of 0.15, and a statistical power of 805%, the minimum sample size was estimated to be 131. Considering the dropout rate, 150 patients were recruited. After excluding data from one participant for incomplete responses, data from a total of 149 participants were used in the final analysis.

The study was approved by the Institutional Review Board of the university affiliated with the authors prior to the start of the study. Data were collected between 15 January and 15 February 2021. At the time of data collection, Korea was under a level 2 social distancing protocol (regional outbreaks, instruction to refrain from unnecessary outings and gatherings, and from using multi-use facilities) due to COVID-19. After obtaining the necessary approval from the applicable institutions and the heads of the dialysis centers, one nurse from each dialysis center was selected as a research assistant for the data collection. The decision to select nurses for this role was based both on the determination that a nurse who is already familiar with hemodialysis patients would be a suitable research assistant and on the desire to minimize the entry of outside personnel to the dialysis unit in order to prevent the spread of COVID-19. The questionnaire surveys were conducted after the head researcher presented guidelines that detailed the purpose of the study, questionnaire content, and survey method to the research assistants at each institution and reviewed the feasibility of conducting the survey in the clinical settings. After explaining the study to the participants, questionnaires were distributed to those who consented to participate in the study voluntarily and submitted an informed written consent form. Participants completed the questionnaire in a self-report format. They were offered the option of completing the survey while receiving hemodialysis or in a consultation room after hemodialysis treatment. The survey required approximately 20–25 min to complete. Participants were gifted a small token of appreciation for completing the survey.

### 2.3. Study Tools

#### 2.3.1. General Characteristics

The survey assessed 12 general items: age, gender, spouse (yes/no), religion (yes/no), education level, employment (yes/no), monthly income, causative disease, duration of hemodialysis, comorbidities (yes/no), things to learn about COVID-19, and source of COVID-19 information.

#### 2.3.2. Burden Due to COVID-19

Burden due to COVID-19 was assessed through a total of 11 items: 6 items focused on burdens of daily life and 5 items focused on burdens of disease management. A numerical rating scale (NRS) was used to rate each item between 0 points (not at all) and 10 points (very much so), with higher total scores indicating higher levels of burden. In this study, the reliability (Cronbach’s α) of the tool was 0.90.

#### 2.3.3. Depression

Depression was measured using a tool translated to Korean, based on the Patient Health Questionnaire-2 (PHQ-2), originally developed by Kroenke et al. [27]. This tool consisted of 2 items, and a 4-point Likert scale was used to rate each item between 0 points (almost never) and 3 points (almost every day). The total score range was 0–6 points, with ≥3 points indicating that the patient “has depression”. In this study, the reliability (Cronbach’s α) of the tool was 0.88.

#### 2.3.4. Awareness of Information about COVID-19 (AIC)

AIC was measured using a tool translated to Korean and modified by the researchers of this study based on the tool developed by Musche et al. [9] for cancer patients. In this study, content validity was assessed by experts (a head nurse of a hemodialysis center, a renal medicine specialist, and an infection control nurse) to judge the content and semantic validity of the instrument after its translation into the local language, Korean. This tool consisted of three items to assess the level of awareness about COVID-19 symptoms, prevention methods, and government policies. A 7-point Likert scale was used to rate each item between 1 point (not at all) and 7 points (very much so), with higher scores indicating higher levels of AIC. In this study, the reliability (Cronbach’s α) of the tool was 0.82.

#### 2.3.5. Safety Behavior

Safety behavior was measured using a tool developed by Musche et al. [9] based on the general recommendations for safety behavior for COVID-19 prevention by the World Health Organization (WHO), which was translated to Korean and modified by the researchers of this study. In this study, content validity was assessed by experts who judged the content and semantic validity of the instrument after its translation into the local language, Korean. This tool consists of eight items: four items on ASB (handwashing, social distancing, changes in meeting schedules, refraining from using public transit) and four items on DSB (hoarding flour, rice, etc.; hoarding hand sanitizers; hoarding toilet paper and other hygiene products; increased selfishness). A 7-point Likert scale was used to rate each item between 1 point (not at all) and 7 points (very much so), with higher scores indicating higher levels of practice of ASB or DSB. The reliability (Cronbach’s α) of the tool for ABS and DBS was 0.74 and 0.77, respectively, in the study by Musche et al. and 0.60 and 0.82, respectively, in this study.

### 2.4. Data Analysis Method

The collected data were analyzed using SPSS/WIN 27.0. General characteristics, burden, depression, awareness of information, and level of preventive behavior were identified using descriptive statistics, including mean, standard deviation (SD), and percentage. The factors influencing ASB and DSB were analyzed using stepwise multiple regression analysis.

## 3. Results

### 3.1. General Characteristics

The mean age of the participants was 58.7 years (range: 23–84 years) and 61.7% (n = 92) of the participants were male. The results also showed that 61.1% (n = 91) of participants had a spouse, 53.7% (n = 80) reported having no religion, 81.2% (n = 121) were high-school graduates or higher, 70.5% (n = 105) were employed, and 36.2% (n = 54) had a monthly income of ≤1 million KRW. With respect to the reason for starting hemodialysis, 49.0% (n = 73) and 40.9% (n = 61) were diagnosed with hypertension and diabetes, respectively. The mean duration of hemodialysis was 68.7 months (range: 2–300 months), with 43.6% (n = 65) of participants on hemodialysis for ≥60 months. Meanwhile, 43.6% (n = 65) of participants reported no comorbidities. Most participants (97.3%, n = 145) reported “TV, newspaper, radio, or other” as the source of information about COVID-19. On being asked what they wanted to learn about COVID-19, participant responses included dialysis catheter management (63.1%, n = 94), mental health management (24.2%, n = 36), handwashing and disinfection methods (24.2%, n = 36), and physical health management (22.8%, n = 34) (Table 1).

### 3.2. Burden Due to COVID-19, Depression, AIC, and Safety Behavior

The mean score for the burden on daily life due to the COVID-19 pandemic was 6.24 out of 10 points, with the highest scores for “restriction on leisure activities” (6.91 points) and “uncertainty about the future” (6.38 points). The mean score for concerns about disease management was 6.76 points, with the highest scores for “concern about exacerbation of disease” (7.41 points), “concern about becoming infected with COVID-19” (7.28 points), and “concern about not receiving hemodialysis on time” (7.07 points). The mean score for depression was 2.05 out of 6 points, with 28.2% (n = 42) categorized in the depression group. The mean score for the level of AIC was 5.26 out of 7 points, while the mean scores for preventive ASB and DSB were 5.77 and 3.05 points, respectively (Table 2).

### 3.3. Influencing Factors of Safety Behavior

To identify the influencing factors on safety behavior, multiple regression analysis was performed with burden (11 items), depression, and AIC as independent variables. The ASB influencing factors were identified as AIC (*β* = 0.265, *p <* 0.001), the burden of “not receiving hemodialysis on time” (*β* = 0.233, *p* = 0.008), and the burden of “social exclusion of hemodialysis patients” (*β* = 0.186, *p* = 0.032), which had an explanatory power of 22% for ASB (F = 13.32, *p* < 0.001). The DSB influencing factors were identified as the burden of “social exclusion of hemodialysis patients” (*β* = 0.258, *p =* 0.003) and AIC (*β* = 0.217, *p =* 0.004), which had an explanatory power of 21% for DSB (F = 12.79, *p* < 0.001) (Table 3).

## 4. Discussion

This study identified the influences of burden, depression, and AIC on safety behavior among hemodialysis patients during the COVID-19 pandemic. End-stage renal disease has a high physical and psychosocial burden on patients who must receive hemodialysis therapy throughout their life until, or unless, they receive a kidney transplant.

In this study, hemodialysis patients showed a high level of burden for disease management, including specific concerns about “exacerbation of disease”, “becoming infected with COVID-19”, and “not receiving hemodialysis on time” due to COVID-19. Burden level was found to influence patient ASB or DSB. The influencing factors of ASB were identified as the burdens of “not receiving hemodialysis on time” and “social exclusion of hemodialysis patients”. Previous studies have reported similar results; anxiety and fear associated with COVID-19 increased adherence to safety behavior among patients with autoimmune diseases and cancer [9,28].

In light of these findings, dialysis unit managers must prioritize the awareness of government policies under the constantly changing pandemic situation and provide a dialysis environment with strict adherence to infection control guidelines. Patients should be made aware of these efforts by the hospitals so that the patients know that they can receive treatment with a sense of safety. Moreover, preemptive infection prevention strategies, including regular screening and early detection of infection-like symptoms by monitoring patients before and after hemodialysis, could help reduce patients’ disease management burdens.

Among the burden items that increased ASB, the “social exclusion of hemodialysis patients” was significant. Interestingly, this item also increased DSB. There is likely a concern that community-dwelling patients with chronic disease could be neglected when medical resources and government policies are reallocated, and support prioritizes COVID-19 patients, patients in intensive care, and healthcare workers during this unprecedented pandemic. Hemodialysis is the only life-sustaining therapy for patients with end-stage renal disease; thus, continuous and consistent healthcare support is essential. Management guidelines and treatment-related institutional upgrades that focus on hemodialysis patients in preparation for an increase in outbreaks of emerging/reemerging infectious diseases are needed.

This study’s findings identified AIC as a factor that increased ASB and DSB. Musche et al. [9] reported that ASB and DSB were higher in cancer patients than in healthy adults under the pandemic conditions when the cancer diagnosis remained the same; AIC was a factor that increased ASB but reduced DSB. Acquisition of accurate information could promote ASB and induce effective safety behavior [29], but the acquisition of inaccurate or indiscriminate information may lead to DSB [15,16]. Most of the participants (97%) in the present study reported that they acquired information through the media, including TV, newspapers, and radio. Generally, mass media provide COVID-19-related information based on relatively healthy adults, and, within that information, a significant proportion of information lacks validity and reliability. Because health-promoting behavior may vary depending on an individual’s ability to select and use accurate information [30], health authorities should cooperate with healthcare professionals to establish a health information network for patients with chronic diseases, such as community-dwelling hemodialysis patients, who are vulnerable to health inequality.

Meanwhile, 63.1% of the participants in this study were interested in receiving educational information about dialysis catheter management. As complications caused by improper dialysis catheter management can be life-threatening [31] and can increase treatment costs, dialysis catheter management is an important issue for patients undergoing hemodialysis. The pandemic may have raised patient interest in dialysis catheter management. Therefore, nurses who manage dialysis and educate patients should assess whether re-education on existing educational content is needed. They must also provide periodic self-care education as conditions continue to change, based on the pandemic situation, and correct patient awareness based on inaccurate information. Administrative departments and medical staff at hospitals must adhere to dialysis unit infection control guidelines and provide information to alleviate unnecessary tension and fear that patients may feel while also preparing plans that include patient participation in infection prevention activities.

In this study, depression was not statistically significant as a factor influencing safety behavior. While direct comparison is difficult due to a lack of previous studies on the relationship between depression and safety behavior, overall study results are likely due to the relatively low influence of depression compared with other variables. Pandemic-induced depression may cause greater distress among patients with chronic disease than among the general public [26,32], and at the same time reduce those patients’ self-care practices and ability to cope with stress [33]. Therefore, future follow-up studies on the relationship between depression and safety behaviors are needed. Medical staff must recognize that depression is inherent in patients undergoing hemodialysis [34] and provide active support to prevent inappropriate safety behavior due to psychological problems associated with a prolonged pandemic environment.

The present study is meaningful in that it statistically confirmed whether hemodialysis patients’ burden, depression, and awareness of information affect ASB and DSB in a pandemic situation. However, the explanatory power of the selected variables was found to be low, at 20–21%. It can be assumed that this is because there are various factors that affect safety behavior other than the input explanatory variables. A follow-up study should be planned to identify factors affecting safety behavior by adding explanatory variables such as individual situational awareness, disease severity [28], public health situation [23,29], health literacy [35], and self-efficacy [36].

## 5. Study Limitations

This study had several limitations. First, since only Korean patients were included in the convenience sampling, the results cannot be generalized. Further research needs to be conducted by diversifying the target region, the size of the dialysis center, and gender. Second, measurements of AIC and safety behavior levels may have been overestimated, as they were calculated based on self-reported responses. Third, the study did not consider the information utilization ability of the participants when identifying subjective AIC. Fourth, the study could not consider all influences based on constantly changing pandemic conditions and government policies (e.g., vaccination protocols).

Despite these limitations, this study was significant in that it identified factors associated with ASB and DSB among hemodialysis patients under the pandemic situation. It produced basic data that can be used for infectious disease control measures for hemodialysis patients.

## 6. Conclusions

In this study, burden due to COVID-19 and related AIC were identified as factors that increasingly influenced the safety behavior of hemodialysis patients. Therefore, based on the reality of the burden reported by hemodialysis patients, healthcare providers need to attempt to implement interventions that alleviate the psychological problems of patients. It is necessary to establish a system to identify the level of COVID-19 information perceived from the perspective of dialysis patients and to correct the misinformation that causes DSB. Additionally, factors that hinder patients in their performance of ASB should be identified, and a system to correct them should be established.

## Figures and Tables

**Table 1:** General characteristics of participants (N = 149).

Characteristics		N (%) or Mean ± SD (Range)
Age (years)		58.7 ± 12.4 (23–84)
	<50	32 (21.5)
	50–59	41 (27.5)
	60–69	47 (31.5)
	≥70	29 (19.5)
Gender	Male	92 (61.7)
	Female	57 (38.3)
Spouse	Yes	91 (61.1)
	No	58 (38.9)
Religion	Yes	69 (46.4)
	No	80 (53.7)
Education level	Junior high school	28 (18.8)
	High school	69 (46.3)
	College graduate or above	52 (34.9)
Employment	Yes	44 (29.5)
	No	105 (70.5)
Monthly income (KRW)	≤1,000,000	54 (36.2)
	1,000,000–2,000,000	37 (24.8)
	2,000,000–3,000,000	25 (16.8)
	≥4,000,000	33 (22.2)
Causative disease *	Hypertension	73 (49.0)
	Diabetes	61 (40.9)
	Glomerulonephritis	26 (17.5)
	Miscellaneous	20 (13.4)
Duration of hemodialysis (months)		68.7 ± 66.2 (2–300)
	≤12	28 (18.8)
	13–59	56 (37.6)
	≥60	65 (43.6)
Comorbidities	No	80 (53.7)
	Yes	69 (46.3)
Things to learn about COVID-19 *		
	Physical health management	34 (22.8)
	Mental health management	36 (24.2)
	Hand washing and disinfection method	36 (24.2)
	Diet	14 (9.4)
	Dialysis catheter management	94 (63.1)
	Miscellaneous	10 (6.7)
Source of COVID-19 information *		
	TV, newspaper, radio, or others	145 (97.3)
	Friend or colleague	8 (5.4)
	Medical staff	3 (2.0)
	Family member	3 (2.0)

* Multiple responses.

**Table 2:** Burden due to COVID-19, depression, awareness of information, and safety behavior (*N* = 149).

Variables	N (%) or Mean ± SD (Range)
Burden due to COVID-19	
Burden of daily life	6.24 ± 2.70
Uncertainty about the future	6.38 ± 3.35
Accumulation of fatigue caused by tension	6.35 ± 3.32
Changes in meeting schedule	5.88 ± 3.33
Declining household income	5.83 ± 3.36
Restrictions on leisure	6.91 ± 3.13
Social isolation	6.09 ± 3.42
Burden of disease management	6.76 ± 2.80
Becoming infected	7.28 ± 3.03
Not receiving hemodialysis on time	7.07 ± 3.43
Shortage of therapeutic drugs	5.89 ± 3.54
Exacerbation of disease	7.41 ± 3.03
Social exclusion of hemodialysis patients (Policies that give priority to the general public and acutely ill patients, etc.)	6.13 ± 3.48
Depression	2.05 ± 1.86
PHQ-2 score < 3	107 (71.8)
PHQ-2 score ≥ 3	42 (28.2)
Awareness of information about COVID-19	5.26 ± 1.53
I feel informed about COVID-19.	5.26 ± 1.75
I feel informed about measures to avoid an infection with COVID-19.	5.36 ± 1.74
I understand the public health authorities’advice regarding COVID-19.	5.15 ± 1.87
Safety behavior	
Adherent safety behavior (ASB)	5.77 ± 1.14
I wash/disinfect my hands more often.	6.03 ± 1.34
I increasingly avoid public places/events.	6.09 ± 1.39
I increasingly avoid public transit (subway, tram, bus, train).	5.53 ± 2.10
I have changed my trip/vacation plans or would change them if I had planned a vacation/trip.	5.42 ± 1.98
Dysfunctional safety behavior (DSB)	3.05 ± 1.54
I have bought larger quantities of basic food (flour, sugar, noodles, rice, and canned food) or will buy more in the near future.	2.66 ± 1.87
I have bought larger quantities of hand disinfectant/soap/similar or will buy more in the near future.	3.71 ± 1.97
I have bought larger quantities of toilet/hygiene articles or will buy more in the near future.	2.83 ± 1.90
I have become more selfish in my behavior.	2.99 ± 1.91

PHQ, Patient Health Questionnaire; ASB, adherent safety behavior; DSB, dysfunctional safety behavior. Answers were given on a 7-point Likert-scale ranging from “1 = strongly disagree” to “7 = strongly agree”.

**Table 3:** Influencing factors of safety behavior (*N* = 149).

Adherent Safety Behavior	B	SE	*β*	t	*p*
Intercept	15.208	1.380	0	11.02	<0.001
Burden: Not receiving hemodialysis on time	0.311	0.115	0.233	2.71	0.008
Burden: Social exclusion of hemodialysis patients	0.245	0.113	0.186	2.17	0.032
Awareness of information about COVID-19	0.264	0.074	0.265	3.59	<0.001
R^2^ = 0.22, Adjusted R^2^ = 0.20, F(*p*) = 13.32 (<0.001)					
**Dysfunctional Safety Behavior**	**B**	**SE**	***β***	**t**	***p***
Intercept	2.763	1.875	0	1.47	0.143
Burden: Social exclusion of hemodialysis patients	0.451	0.164	0.258	2.99	0.003
Burden: Changes in meeting schedule	0.314	0.172	0.170	1.83	0.070
Awareness of information about COVID-19	0.290	0.099	0.217	2.92	0.004
R^2^ = 0.21, Adjusted R^2^ = 0.19, F(*p*) = 12.79 (<0.001)					

## Data Availability

Data are available upon request.
